# Intra-rater and Inter-rater Reliability of Hemoglobin Color Scale Method

**DOI:** 10.4103/0970-0218.58405

**Published:** 2009-10

**Authors:** SB Agampodi, MSM Kularathna, PMRBI Pathiraja

**Affiliations:** Department of Community Medicine, Faculty of Medicine and Allied Sciences, Rajarata University of Sri Lanka, Sri Lanka; 1 Department of Community Dentistry, Faculty of Dental Science, University of Peradeniya, Peradeniya, Sri Lanka; 2 Department of Community Medicine, Faculty of Medical Sciences, University of Sri Jayewardenepura, Nugegoda, Sri Lanka

Sir,

Anemia during pregnancy is a major public health problem in Sri Lanka as well as in other developing countries. The clinical method of detecting anemia is having a low accuracy.(1,2) The National Maternal and Child Health Program – Sri Lanka, has introduced the Hemoglobin Color Scale (HCS) to assess Hb levels in field clinics since 2007. The HCS test has been developed by the WHO, as it is a simple, reliable, and inexpensive method of diagnosing anemia when laboratory methods are not freely available.(3) The objective of the present study is to test the intra- and inter-rater reliability of HCS tests in the actual field setting.

We conducted the present study in Kalutara district, Sri Lanka. Five randomly selected pregnant mothers were invited for the study. Five public health midwives (PHMM) from the field were selected randomly and invited for the study. We used a balanced randomized block design, with repeated observations, for reliability testing. A single mother was tested using five strips. Reading of Hb values were done by PHMM who were blinded for the identity of strips. Five Hb strips representing all five subjects were given to each PHMM for recording of HB values. Strip sets were exchanged and reread by different PHMMs. The same two sets they already observed were given to them for a repeated reading without their knowledge.

One hundred readings were obtained in all. A single PHMM recorded four readings [two repetitive readings] for a subject, thus 20 recordings for all five subjects. Readings were completed within five minutes. Percentage agreement between two readings for an individual PHMM varied from 20 to 90%. Table 1 shows the various measures of intra-rater reliability.

**Table 1:** Intra-rater reliability measurements for individual PHMM

PHMM	Variance	ICC for repeated measurements	*P*
A	0.789	0.757	0.004*
B	2.221	0.301	0.184
C	1.042	-0.408	0.894
D	0.789	0.757	0.004*
E	1.053	-0.6	0.974

Variance of the repeated reading shows wide variation, that is, 0789 to 2.221 between PHMMs. Only two observers had very good Intraclass Correlation Coefficient (ICC) (0.757), which was highly significant (*P* = 0.004). One observer reported non-significant poor correlation (ICC 0.301, *P* = 0.184), while two remaining observers reported no correlation at all between repeated readings.

A single strip had four readings. Descriptive analysis of strips yielded that only 4/25 strips (16%) had 100% concordance, while 14 (56%) had 75% concordance rate.

ICC for test / retest reliability for strips was 0.162, where the correlation was very poor and not significant (*P* = 0.699). Repeated measure analysis of variance (ANOVA) showed highly significant differences between observers (F = 10.05, *P* < 0.001) and negative ICC value (- 0.047), which indicated that there was no correlation at all between the observers.

Our study showed that the inter-rater and intra-rater reliability was very poor for HCS, among our study sample. However, previous studies have shown that the HCS test has high reliability and validity.(4) The present study was conducted in a natural field setting, during their routing procedures, to resemble the actual environment where they usually apply this test. Following of standard procedure was not observed because our prime objective was to assess the present practice. These limitations might have an impact on our observations.

Retraining of PHMM in a standard procedure and identification of PHMM with reliable strip reading capabilities and allocation of the task only to those, would help to overcome this problem. However, the study was a preliminary study. Generalization of the findings cannot be done due to limitation in the study. A study with properly calculated sample size, with an adequate number of pregnant mothers representing a wide range of HB levels is recommended.
